# Why too much biomedical research is often undeserving of the public’s trust

**DOI:** 10.3389/fgene.2025.1587616

**Published:** 2025-06-26

**Authors:** Mark Yarborough

**Affiliations:** Division of General Medicine and Bioethics, Department of Internal Medicine, School of Medicine, University of California Davis, Sacramento, CA, United States

**Keywords:** trust and trustworthiness, research quality, research ethics, research regulation, trustworthiness of research

## Abstract

This article queries whether the public can be reasonably confident that the biomedical research endeavor repays the public’s trust in it with research that routinely deserves that trust. I argue below that a research endeavor that would deserve trust is one that routinely produces research whose published results are dependable, investigates socially important questions, and is conducted ethically. While various inferences can be drawn about terms like “routinely,” “dependable,” and “socially important,” I think they are still informative enough to fruitfully guide the query that follows. The query is shaped by two stipulations that are explicated further below. The first is normative: a collective endeavor that enjoys a broad range of public concessions, such as government funding, favorable public policy like patent law or tailored legal immunities, or widespread support from private philanthropy, all meant to facilitate the endeavor, ought not solicit the public’s trust that gives rise to these concessions without being confident that it deserves it. The second is that confidence requires effective and transparent accountability. The query concludes that the public cannot be reasonably confident that the biomedical research endeavor routinely repays the public’s trust in it with research that deserves that trust^1^. A final item of note about the query is that it does not directly engage the recent Covid pandemic. The reasons it does not are that there is already ample engagement around that episode on the one hand and, on the other, the items of concern that are addressed in the query long predate that particular pandemic and the controversies it has engendered, many of which will likely persist no matter what eventual reforms might follow from the resolution of Covid-specific controversies.

## Introduction

Our query begins with a quote from Paul Gelsinger. In 2001, a little short of 2 years after the death of his son, Jesse, he wrote that, “All research subjects really want is to be able to trust the system.” (Paul Gelsinger) His son had volunteered for a Phase 1 trial of a novel gene therapy for a rare genetic disorder he was affected by. Paul Gelsinger’s written account followed his disillusionment with what he came to understand about the research oversight system subsequent to his son’s death. His son died a few days after he was transfused with the experimental agent, making him “the first person ever publicly identified to have died in a clinical trial for gene therapy” (Jesse’s legacy, 2011).

His son volunteered for the trial in hopes of helping others, like him, born with ornithine transcarbamylase deficiency (OTCD), which is a rare and sometimes fatal metabolic condition that causes ammonia to accumulate in the blood. If severe enough, it “can cause liver and nerve damage, lethargy and coma” (Jesse’s legacy, 2011). Though Jesse had occasional severe bouts from his OTCD growing up, it was eventually well controlled by medication, so much so that he was living a quite active and normal life at the time he decided to enroll in the Phase 1 trial. Eight days after that fateful decision, he was dead, never again having the chance to speak with his family and friends after his departure from his home in Tucson to Philadelphia, the site of the clinical trial.

Paul Gelsinger reports giving his son a big hug at the airport and exchanging “I love yous” only to have to rush to Philadelphia a few days later, where he found his son unconscious and dying from “a massive immune response to the virus that was a component of the trial’s injections.”

Repercussions from Jesse’s death were wide-ranging, first encompassing the lead investigator on the ill-fated study but eventually extending to the biomedical research community at large. Following a 5-year investigation by the United States Department of Justice, the lead investigator was subject to 5 years’ worth of sanctions related to the conduct of clinical research. The University of Pennsylvania, which had a major financial stake in the biotech company developing the gene therapy, had to pay a $500,000+ payment to the federal government. The Food and Drug Administarion (FDA) and National Institutes of Health (NIH) “tightened monitoring of trials, increased inspections, and created a new system for reporting serious side effects” for gene therapies (Science History Institute, 2019). Lastly, Jesse’s death contributed to efforts to better manage financial conflicts of interest in research, which have largely taken the form of new disclosures by researchers of their financial relationships (Office for Human Research Protections, 2016; Basken, 2010).

Yet, despite these and other reforms, Paul Gelsinger remained deeply skeptical of clinical research oversight. In a quote 10 years after his son’s death, he stated, “I never will trust the system again [because t]he system’s not trustworthy yet.” (Jesse’s legacy) More than the passage of time and the enduring pain of the loss of his son’s life accounts for this statement. During that interim period Mr. Gelsinger had been both party and privy to several efforts to reform research and research oversight that resulted from litigation following his son’s tragic death. Thus, his 2011 statement emanated from a place of greater familiarity with and insights about the world of biomedical research, indicating there is much more about the biomedical research system than his son’s fatal encounter with it that shapes his indictment of it.

Is his indictment unfair? As someone who has been involved in biomedical research and its oversight for several decades, I wish I could say it is. However, having also spent many of those years asking whether the system is trustworthy, I must confess at this point that I have concluded that his indictment is warranted. This essay is meant to give readers the opportunity to learn both about the system and why I think it is too untrustworthy, so that, now more than 20+ years after Mr. Gelsinger’s son’s death, they can ask themselves whether “the system is trustworthy yet.” To productively ponder the question, we need a framework to direct our reflections and considerations about it.

## What might a trustworthy system look like?

Critical to our efforts to examine the extent of trustworthiness in biomedical research is an examination of the characteristics that warrant research being designated as trustworthy. We start, though, with the topic of trust itself, as well as common invocations of it, in the context of biomedical research.

### A few words about trust

Trust is ubiquitous in our lives. It is part of the very fabric that makes social intercourse possible. Accordingly, a rich and diverse body of work has emerged that provides various accounts of trust in an attempt to understand it. I would refer readers to such scholars as Onora O’Neill, Annette Baier, Russell Hardin, and Francis Fukayama, to name only a few.

There is also a substantial body of work that examines trust in the expansive clinical context of medicine. Scholars such as Giselle Corbie-Smith, Mark Hall, Susan Goold, and Susannah Rose are but a few of the talented individuals who have helped to characterize and measure trust in the context of medical care, healthcare organizations, and clinical research. When one looks at scientific research in general and biomedical research specifically, Richard Smith, former editor of *BMJ*, reminds us that science “depends wholly” on trust (Smith, 2014). In a similar vein, philosopher John Hardwig has shown that scientists’ trust in other scientists is a precondition for scientific knowledge itself (Hardwig, 1991).

### Trust and trustworthiness

This essay, while inspired by much of this rich work, does not aim to further plumb either trust itself or the specific ways that it contributes to scientific investigations. Instead, it has a different focus, which is to look at trustworthiness so that we can examine the frequency with which it is found in the context of biomedical research. To do this, we will need to plumb research more than we plumb trust. Being able to answer questions about the trustworthiness of research requires an understanding about what research is supposed to do. Only once we know what research is supposed to do can we hope to answer the question, “can it be trusted to do it?”

Before turning to that question, however, there are some considerations about trustworthiness that we need to bear in mind. The first of these is that, even though trustworthiness is distinct from trust, they both nevertheless go hand-in-hand and it is difficult to talk about one without having to talk about the other from time to time. Thus, I will still occasionally need to reference trust in research when I interrogate its trustworthiness.

Second, when we do go back and forth between trust and trustworthiness, it will be important for readers to keep the following in mind: trust is a state of mind, perhaps even a state of being-in-the-world and there is neither a set of facts about the world nor words uttered by others that can compel one to either adopt or change that state of mind. Trustworthiness, on the other hand, refers to tangible matters. A thing or endeavor either does or does not possess the properties that confer trustworthiness upon it. Others have also noted how trust is far from synonymous with trustworthiness (Ryan, 2020; Hall et al., 2001; Neill, 2002). “Patients can misplace trust in physicians or institutions that are not deserving, or they can fail to trust those that are.“ (Office for Human Research Protections, 2016) “Deceivers can attract others’ trust [while] the trustworthy can be denied others’ trust” (Basken, 2010).

Thus, we need to keep this dichotomy in mind in what follows because many people are disposed to extend their trust to research, for any number of reasons, and it may be virtually impossible to shake their faith in the scientific project. Similarly, other people will be disposed to withhold their trust from research, almost as a reflex, again for any number of reasons that are beyond the influence, let alone control, of the scientific community.

Unfortunately, this dichotomy often goes unappreciated within the research community, which has contributed to a tendency within it to pay far more attention to being trusted rather than being trustworthy. Consider the words of Francis Collins, former Director of NIH. He opined that “[t]he public trust in [biomedical research] is just essential, and we cannot afford to take any chances with the integrity of the research process.” (Wadman, 2010) The occasion for his words was adverse publicity that ensued following public accounts about undisclosed financial ties between a leading academic psychiatrist and the drug companies that funded his research (How An Ethically Challenged Researcher).

The then NIH Director’s sentiments mirrored a lead editorial in *Nature* that same year. It opined that “cultivating the public’s trust must be the scientific community’s top priority (Nature, 2010). The occasion for this pronouncement was ongoing controversy about “Climategate,” which ensued following public revelation of climate researchers’ hacked emails. Though multiple investigations all found that the emails revealed little if anything of concern about the researchers and their research, to the lay eye the emails raised suspicions that data were being manipulated and falsified by researchers.

There has been no shortage of research scandals, accompanied by yet more pronouncements about the need to better guard the integrity of research, since these pronouncements were made in 2010. Recent examples of scandals include resignations of the leaders of two of the leading private universities in the United States, Harvard and Stanford, due to longstanding indifference to, if not willful violation of, ethical standards of research (Claudine Gay Resigns as Harvard; Saul, 2023). Nor must we fail to note that there were ample scandals well prior to 2010, also accompanied by public pronouncements and reforms, that similarly failed to achieve the desired results.

One obvious reason for this disappointing track record may well be that the well-intended pronouncements and reforms disproportionately occur in response to scandals, reflecting a reactive as opposed to a proactive stance toward the public’s trust within the research community. Perhaps a less obvious reason the research community focuses on trust, when it does, is because it may care more about enjoying the public’s trust than it does deserving it. How else should we account for the fact that it is when the benefits of being trusted are perceived to be at risk that the public’s trust garners the most attention within the research community? And how else might we account for the fact that when furor subsides, the largely complacent *status quo* ante resumes?

Both of these likely reasons suggesting why the research community is more reactive than proactive about the publics’ trust are deeply problematic. First, if one agrees that the public’s trust is “just essential” to research, then the prudent course of action for the research community would be to be laser-focused on that trust continuously, not sporadically, and especially not just in the wake of scandal.

Of greater concern about this track record, however, is that it suggests that the public’s trust warrants (only sporadic) consideration because what only matters is enjoying it. This leads to curiosity about learning how best to manufacture it so that it can be enjoyed, not about whether the public’s trust is truly deserved in the first place. A recent example of this is a NIH funding opportunity, “Build Up Trust,” that has a heavy emphasis on increasing public participation in biomedical research (NIH Build UP Trust Challenge).

When the research community is motivated to just enjoy the public’s trust regardless, then the concern deepens because it is a signal that the research community is satisfied to enjoy trust in all its guises, whether it be deserved, earned, misplaced, or undeserved. Enjoyed trust comes in all of those varieties and a research community that only cares that it enjoys trust will never take upon itself the additional burdens of learning which flavor of trust it is enjoying at any point in time, nor enjoy any of the improved quality in research that those burdens would spawn.

While I do not want to lay all these interconnected problems at the feet of failing to appreciate the dichotomy between trust and trustworthiness, or being trusted and being trustworthy, one cannot help but believe that these problems would be less likely to arise if the research community noted the dichotomy and took its implications to heart. One way it could do so is to adopt a postulate that I have discussed elsewhere (Yarborough, 2021a). That postulate is that a moral precondition of every solicitation of the public’s trust is being confident that one is deserving of it. In support of this postulate is the advice of Russell Hardin, one of the noted trust scholars I mentioned above, who says that the key to being trusted is deserving to be trusted (Hardin, 2002).

In defense of this postulate, consider an analogy. All of us would quickly condemn the solicitation of charitable contributions by an organization that uses the solicited funds for undisclosed purposes, or that was incapable of achieving the purposes promised in its solicitations, or that was incurious about the effectiveness of its charitable work. Such an organization would be unworthy of the trust it was asking from its donors. While biomedical research is not exactly analogous to a charitable organization, I think it is close enough to one that we would similarly disapprove of solicitations of trust by the research community if it were either incapable of or incurious about delivery on its promises. Even if one is unwilling to grant my postulate, I suspect most would agree that, since the research community continuously solicits the public’s trust, it should be curious to know to what extent and with what frequency it deserves the trust it is soliciting.

Instead of such curiosity, what we find instead is deeply troubling. Despite the presence of various research ethics codes, professional practice norms, and laws and regulations pertaining to biomedical research, there is no consensus within the research community about what it is, exactly, that makes research deserving of the public’s trust. Consequently, one also searches in vain for consensus about what practices promote trustworthy research and how those practices are best assessed and improved. This uncertainty matters for both reasons of prudence and ethics. It matters for reasons of prudence if one takes at face value proclamations like the one from Francis Collins. It matters for reasons of ethics if one agrees with the postulate that one ought not solicit another’s trust if one is not confident, or at least curious to know whether, that trust is deserved in the first place.

How does one move forward, then, and do both the prudent and ethical thing in this unclear context? Progress will require us knowing, exactly, what things the research community should rightly be trusted to do and how it goes about routinely doing those things. It also means that so long as we lack consensus about the things the research community should rightly be trusted to do and how it goes about routinely doing those things, we will remain reliant on the current *status quo* that, I fear, leaves the public’s “essential” future trust to chance rather than design (Yarborough, 2014a).

Others may be more sanguine about the *status quo*, given the extent of current research safeguards that have been spurred by past reform efforts. Besides the numerous international statements and professional codes stipulating what makes biomedical research ethical, there are professional norms, reflected in the international statements and codes of ethics and transmitted in science education; quality control mechanisms such as peer-review; research regulation activities like Institutional Review Board (IRB) and other research ethics review; and a growing global reliance on both research integrity programs and mandatory instruction in the responsible conduct of research. Though all of them aim to protect biomedical research, there is ample evidence, of which only a small representative sample is discussed in this essay, showing that we should question the effectiveness of these safeguards and why supplementing them with a persistent focus on deserving trust could help usher in a new *status quo*.

## A conceptualization of trustworthy biomedical research: three criteria and their justification

That new *status quo* will need to be firmly embedded in the recognition that there is such a thing as deserved trust. Consider a simple, fundamental question, which is “why should I trust a researcher or their research?” Answering it begins by noting both that research comes with all manner of attributes and characteristics and that we are discriminating in our assessments of any given instance of research based upon those attributes and characteristics.

Before turning to what these multiple attributes are, I first need to note here that referencing multiple attributes of trustworthiness is at odds with other views. To cite just one example, some scholars, following common parlance for the term “trustworthy,” equate trustworthiness in research with reliability (Strech et al., 2020), reflecting the commonplace tendency in the research community to conflate trustworthy research with reliable research.

While this view is certainly defensible, that defense exacts a price. The benefits of any self-imposed obligation to deserve the trust that gets solicited would be confined to those matters pertaining to research reliability. Though, as we discuss later, such matters as methodologic rigor and accurate reporting are vital to the very conduct of science itself, when we equate trustworthiness with reliability alone, we commit ourselves to the view that the research community could rightly deserve the public’s trust so long as certain methodologic attributes are present in investigations, regardless of *what* is being investigated and regardless of *how* it is being investigated. Few would contend that the public’s trust should be indifferent to these other aspects of research.

To further appreciate why relegating the realm of trustworthy research to the realm of reliable research is too parsimonious a view to hold, consider an analogy from engineering. Asking what makes a bridge, for example, trustworthy would quickly elicit responses along the lines of it is safe to traverse. But surely the legitimate expectations we collectively have about the bridge extend beyond the expectation to be able to use it to safely cross from Point A to Point B. Even though we prize the ability of engineers to construct a bridge that we can use to safely travel between two points, just as we prize the ability of researchers to produce reliable information, there is much more to the story that relates directly to trustworthiness. In order to achieve the construction of a safe bridge, there are additional considerations that must be taken into account, as is equally true in the research context.

Consider the once infamous “bridge to nowhere” meant to connect Ketchikan to Gravina Island, Alaska, in the United States, even though its population was roughly only 50 (National Public Radio 2008). The importance of being able to get from Point A to Point B must be sufficient to offset the costs incurred by its construction. In other words, it has to be worth it to build the bridge, which is comparable to the need we find in biomedical research that investigations must meet some minimal threshold of social worth. Further, there are limits to the environmental degradation that a bridge should be permitted to cause. Similarly, there are ethical norms that should impose comparable constraints on biomedical research. If there is merit to this analogy, then there are multiple attributes of trustworthy biomedical research that can be derived from the collective expectations that surround it. So too, there are ways of learning the frequency and extent to which those expectations are met.

The engineering analogy shows why trustworthy research is not synonymous with reliable research and why there are at least three characteristics that make biomedical research trustworthy: dependability, rather than reliability as I explain below; social value; and ethical conduct. Let us look at each.

### The role of dependability in making biomedical research trustworthy

In previous articles I have used the term reliability to refer to one of the key attributes of trustworthy research. I used that term as a kind of catch-all phrase for multiple audiences, including lay ones, meant to establish that we should be able to routinely trust research reports to convey information that others can depend upon to either enhance their understanding of a topic or inform subsequent research. However, reliability also has a more technical and thus limited meaning as well, and it quickly gets caught up in discussions within the research community about equally technical and important terms that all relate to the ability of others to depend upon the information that researchers disseminate about their work. These terms include replicability, repeatability, reproducibility, validity, robustness, and accuracy, all of which have both general and precise meanings. To help illustrate this, consider the following written by some commentators: “Scientific results should not just be ‘repeatable’ (replicable in the same laboratory under identical conditions), but also ‘reproducible’ (replicable in other laboratories under similar conditions). Results should also, if possible, be ‘robust’ (replicable under a wide range of conditions).” (Roper et al., 2022) Thus, we see that research might be “repeatable” but not “replicable” and “reproducible” but not “robust.” Similarly, results may be “accurate” in one context but not another, due to precise technical definitions often attached to them. However, unless it is important to bring out these subtle, and in some contexts, critical distinctions, I think it is helpful in broad discussions about trustworthiness in research like this one to find a term that can capture the importance of all of these research attributes, even though the practical realities of research, such as budgets, lab settings, modeling and the like often make it impossible for any single experiment to possess all of the attributes.

I think the preferred term is dependable. It conveys the key general point that trustworthy research reports are ones that others can depend upon to understand the reported research and its limitations so that it can inform future investigations, analyses, and the like. Also, since dependability is used less frequently in discussions about trust and trustworthiness in research than reliability is, it avoids the problem that reliability has when it gets treated as synonymous with trustworthiness.

The justification for including dependability as a key characteristic of trustworthy research comes from the nature of scientific activity itself. Scientific research emerged as the current social practice it is in part because, for any number of reasons, having “justified” as opposed to “mere” belief frequently matters and scientific research has shown time and again how remarkably adept it can be in establishing such beliefs (Douglas, 2009). We are able to depend upon it to produce such beliefs due to *how* it is done. This shows both that methods matter deeply in scientific research and that researchers must have a strong fealty to those methods. A failure to employ and/or abide by appropriate methods would result in an inability to produce and disseminate dependable knowledge about natural phenomena frequently enough. Too frequent lapses in fealty to proper methods could lead the public to eventually realize that their trust in research is misplaced.

### The role of social value in making biomedical research trustworthy

Given the expense of many of the methods in 21st century science, most of the science enterprise is contingent upon others being willing to pay for it. As others have noted, “science and society [are] joined by an informal, unstated ‘social contract’ – where society permits (and provides support for) the scientific community, and the scientific community agrees to conduct meritorious studies that will contribute knowledge” (Meslin and Cho, 2010).

Talk of an “unstated contract” in support of “meritorious studies” also elucidates that, at least in the case of biomedical research, there is a *why* to research that is just as critical as the *how* of research. When leaders of federal research organizations such as the NIH ask for taxpayer money and when pharmaceutical, biotech companies or even individual researchers solicit clinical trial volunteers, these asks are accompanied by a promise, either implicit or explicit: requested support will be used for investigations that hold the potential for producing information that might prove useful to efforts to promote human wellbeing and/or improve healthcare.

This reveals a frank transaction, or *why*, at work in the case of biomedical research (Mildred Cho, PhD, private communication). Thus, while no one should claim that biomedical research must produce, e.g., a cure for cancer, biomedical research should be able to be trusted to produce information that contributes rationally to such an effort. In other words, biomedical research is meant to seek knowledge in pursuit of discrete, pre-specified goals (WMA WMA). If there is no rational connection to be made between the research in question and the production of knowledge that relates to pursuing such pre-specified goals, then that research is of no value to the transactions that made the research possible in the first place; nor would it deserve anyone’s trust that was extended on the basis of the pre-specified goals.

### The role of ethical conduct in making biomedical research trustworthy

Today’s biomedical research, by its very nature, can be dangerous and exploitative since its conduct necessitates using people, other animals, biospecimens, and/or private information. This reveals that the *manner of conduct* of biomedical research is just as critical as is its *how* and *why*. While there are ample lessons from history showing how there can be a dark side to biomedical research, we would be within our collective rights today to guard against nefarious outcomes even if we had a pristine history as our guide instead. Such a history would not place our critical rights and interests at less risk and we would still be wise to write and enforce such documents as the Declaration of Helsinki (WMA). Such documents set forth international legal and ethical standards for the manner of conduct of biomedical research and reflect the consensus that researchers are not and should not be left to their own devices when conducting their research. Collectively we have decided that it is in all our interests to place constraints on the conduct of research. If the public could not trust these constraints to hold, the entire biomedical research enterprise would (eventually) prove unsustainable.

There may well be additional attributes of research that similarly contribute to its trustworthiness and the research community would surely benefit if further discussion about the attributes of trustworthy research ensued. For this to occur, however, the research community would need to shift away from both its current reactive approach to the public’s trust toward a more proactive one and away from a preoccupation with enjoying the public’s trust toward deserving it instead.

## An overview of the system that people are being asked to trust

With a conceptual framework for interrogating the trustworthiness of the biomedical research system in hand, we can now look to critical features of the system itself that frame the experimental pursuits within it. Our observations begin at an altitude of about 35,000 feet. From this vantage point, readers might expect the standard description of the “drug pipeline” that starts at the bench and ends at the bedside. That oft-used recitation is not quite right though. Instead, the observations herein are informed by Jonathan Kimmelman and Alex London, who write that “[t]he so-called drug pipeline is not really about drugs and is not much like a pipeline.” (Kimmelman and London, 2015) Instead, they contend that the clinical translation effort from bench to bedside is best understood as being “about the production and dissemination of information, and it is much more like a web [that captures] information about the coordinated set of materials, practices, and constraints needed to safely unlock the therapeutic or preventive activities of drugs, biologics, and diagnostics.” (Kimmelman and London, 2015) Thus, throughout what follows I will be assuming that the goal of translational biomedical research is the creation of a rich information ecosystem capable of showing us how to develop a range of interventions, be they drug, device, surgical, or behavior-based, that can be used to prevent and ameliorate the consequences of human disease and disability.

I think this shift away from the “pipeline” metaphor is warranted because so long as our focus in on what comes out of the pipeline, I worry that we set the system up for certain failure. There is the very real prospect that the pipeline “stuff” will never work the way we hope it will. We may never have a magic pill or implantable device that wipes away, let alone prevents, the ravages of Parkinson’s, many cancers, or chronic diseases like diabetes, simply because of the complexity of people and how they interact with and are affected by all the social, psychological, biological, ecological, behavioral, bacteriological, and myriad other variables that affect them continuously. Thus, if we judge the success of biomedical research by the production of magic bullets, we impose an unachievable outcome upon it. It is better instead to adopt a metric more closely aligned with what the endeavor actually does, which is produce information of the sort described by Kimmelman and London for the purposes they note.

With this clarity about the actual landscape we are surveying from 35,000 feet in mind, we can now look to the various stages and types of information production. What we see is a production system that starts by looking for promising ideas, at the basic level of research, that may warrant further and more precise investigation in preclinical studies. When those studies show sufficient promise, they warrant further refinement and possible eventual investigations in the clinical setting, in hopes that the information derived from those clinical trials can reliably guide future clinical treatment decisions and options.

**FIGURE 1:**
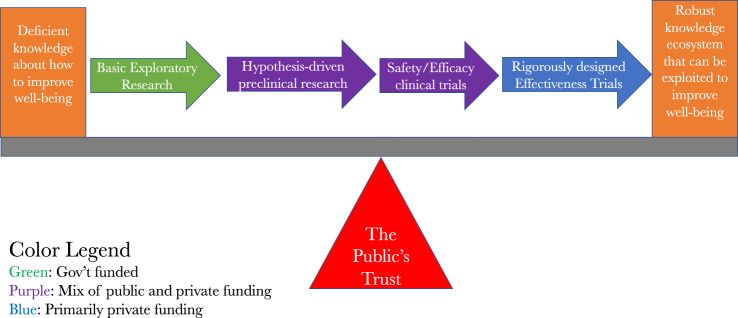
The path to clinical translation.

Figure 1 is a fairly standard way of capturing this, except it has incorporated the insights of Kimmelman and London and it has an added financial overlay on it to indicate where the sources of funding for any particular stage is most likely to come from. It shows that, for the most part, it is the public that pays for the basic and preclinical insights that lead to clinical investigations and it is private companies that mainly pay for the clinical investigations. Patents and other intellectual property rights are assigned along the way, which, depending upon the predilections of the owners of them, can be used to expand or restrict access to the fruits of the largely publicly funded seminal discoveries. Given the outsize role of public funding of the basic discoveries that lead to clinical investigations, combined with the necessity of human volunteers for the clinical investigations, we also see how the public’s trust serves as the fulcrum for the clinical translation effort.

**FIGURE 2:**
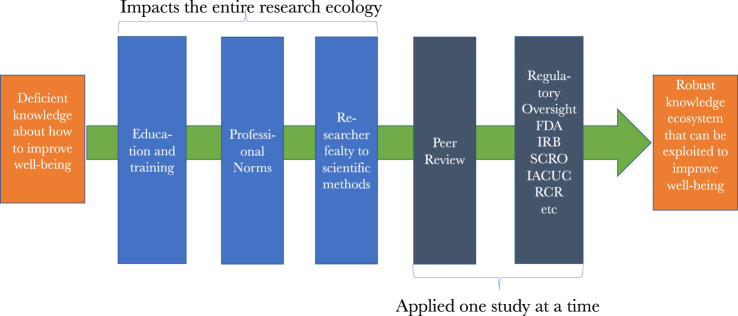
Safeguards along the path to clinical translation.

Figure 2 represents the numerous safeguards that are overlaid on research along the path to the robust knowledge ecosystem that we are pursuing. The most prevalent are the previously mentioned education, training, mentoring, and exposure to professional norms that researchers are subject to as they advance in their careers. Each of these impart critical knowledge, skills, values, commitments, behaviors, and beliefs that shape researchers’ work at the various points in the ecosystem of discovery. As investigators pursue funding and publications, then peer review gets added to the oversight picture. Once we reach studies using certain animals or stem cells, as well as most every study involving human subjects, then safety, e.g., from biohazards, and ethics review is added to the picture. When licensure for drugs or devices is sought, then review by regulatory agencies such as the FDA occurs. These reviews, as well as peer review, occur sequentially, making oversight both continuous and, at times, redundant, which strengthen the protections and assurances the system is designed to afford.

**FIGURE 3:**
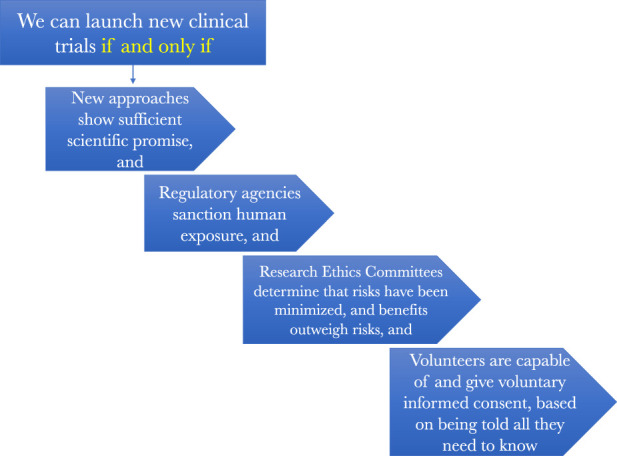
How the regulatory oversight system is supposed to work to assure scientifically and ethically sound research.

Figure 3 presents a more in-depth picture of the regulatory oversight that is meant to assure that early phase clinical trials of the sort that Jesse Gelsinger volunteered for get conducted ethically. What is worth noting from it for our purposes is that clinical trials should proceed only when there is a robust enough body of scientific evidence showing that there is both sufficient eventual therapeutic prospects for testing something and that it can be tested relatively safely enough in those first subjected to it. In other words, there must be a robust enough body of evidence that permits us to make inferences about the prospects of both safety and potential eventual health benefits.

Knowing whether this threshold of sufficient scientific promise that can be safely tested is a task reserved primarily for research ethics committees, such as IRBs. They must judge that there is a reasonable balance between these prospective benefits, in the form of an enriched information ecosystem, that will accrue to society by conducting the clinical research and the risks the research will impose on the humans who participate in it.

Finally, clinical investigations should proceed only after one or more ethics reviews also determine that the people who are candidates for the clinical studies, or their surrogate decisionmakers, will be given all the information they need to be able to decide for themselves if they want to be one of the objects of scientific investigation in a particular study. Only when they are provided this information, ponder it, and decide that participation makes sense for them can they become human subjects in clinical research. This was the system in place in the United States at the time of Jesse Gelsinger’s death and it is the system that remains in place for comparable trials today.

If Mr. Gelsinger’s 2011 claim that the system was not trustworthy is mistaken and the system is in fact trustworthy rather than untrustworthy, then the people who are approached about volunteering themselves or their loved ones for a clinical trial will routinely be able to trust that the various components of the system reflected in the Figures actually warrant their trust. Some additional groundwork is still needed before one could confidently conclude this, however. First, we need to look, again from the 35,000 level, at what the system captured in Figure 1 is producing, which we turn to next.

## What the system is producing: the pursuits and fruits of biomedical research in context

One need not ascribe to David Hume’s view that ethics derives from the moral sentiments to believe that humans care deeply about the suffering of others. Arguably, it is this broadly shared view that makes our current biomedical research endeavor, embodied in the system briefly explored above, possible in the first place. Absent it, we would be hard pressed to account for the significant public dollars devoted to research each year and the financial support extended to the educational institutions that train those who carry it out.

Nor would it be easy to account for the large numbers of charitable organizations that count biomedical research among their missions, the countless public fund-raising campaigns, such as telethons, marathons, walkathons, and direct solicitations for money, such as those at grocery store payment terminals in check-out lanes, that all direct generous additional financial resources to research.

Absent the shared sentiment, there would also be gaping holes in the mission statements of research organizations such as NIH, whose mission is to. “Enhance health, lengthen life, and reduce illness and disability” (National Institutes of Health, 2014) and the American Association for the Advancement of Science, whose mission is to “advance science, engineering, and innovation throughout the world for the benefit of all people” (AAAS, 2025).

This same sentiment is also echoed routinely by many researchers when they relate why they chose careers in biomedical research. People like me who have had the privilege to teach biomedical researchers who are students or early in their careers know how commonplace stories among learners are about being motivated to do research because they had been witness to the suffering and death of a cherished grandparent, parent, sibling, or close friend due to an intractable illness.

It is this same sentiment that surely also helps to explain the significant public trust, reflected in public polling, that was previously extended to science and scientists, and by extension biomedical research, across the globe (Funk et al., 2020b). Though public trust had long been waning in many of the government bodies and agencies, professions, and institutions vital to a vibrant society, trust in the scientific community was a notable outlier among otherwise increasingly skeptical publics. In the United States, public polls showed that the public’s trust in the scientific community was remarkably stable over the last several decades and that biomedical researchers fared better than did scientists in general, military leaders, clergy, and several others (Funk and Kennedy, 2020; Funk, 2020). Though degrees of trust varied somewhat across ethnic groups, (Funk et al., 2020a; Funk, 2022), there was a reservoir of public good will that helped to sustain biomedical research. Especially worth noting is that this reservoir was initially only slightly diminished by the Covid pandemic, with 78% of the US public reflecting either a great deal or fair amount of trust in medical researchers in December 2021, compared to 87% in January 2019 (Kennedy et al., 2022). More recent polling results show that public confidence in medical research has since increased such that it approximates the pre- Covid levels (PNAS, 2025).

These related considerations all reflect our shared understanding that suffering the premature death of a loved one is a tragedy, one made worse when it is accompanied by a chronic and debilitating illness where we witness their lives gradually be diminished by the ravages of disease, which in turn helps to account for the prized standing of the biomedical research endeavor in the eyes of the public.

This ongoing reservoir of support has paid countless public health and clinical care dividends, reminding us that even if the system is not sufficiently trustworthy, it nevertheless manages to still work well at times. Looking just at the drugs that the research information ecosystem has made possible, these dividends come in the form of both lifesaving and life-improving ones. Antibiotics often mean we no longer live in fear of life-threatening infections like our ancestors did. Children who used to all die from pediatric leukemias now routinely survive them. Drugs have similarly transformed HIV from a fatal disease to a chronic one that affords most HIV + people who have access to care full and rich lives. Immunosuppressive drugs, combined with advances in anesthesia and surgery, have led to organ transplants that secure the survival of thousands of patients who otherwise would have died from organ failure.

Drugs such as Spinraza (nusinersen), (SPINRAZA^®^, 2025) which has turned the previously untreatable and lethal variants of spinal muscular atrophy into a treatable condition, show that biomedical research can indeed hit home runs. Immunotherapies are producing similarly impressive results for some cancer patients. There is now fervent hope, built upon genuine progress, that gene therapies, which have now largely recovered from the stigma that was attached to them after Jesse Gelsinger’s death, will similarly alter the life course of people with heretofore devastating and often untreatable genetic diseases such as sickle cell, Huntington’s disease, and countless other life-limiting genetic conditions. A major portion of this hope is being fueled by new technologies like CRISPRcas9, human organoids, organ chips, and artificial intelligence employing large language models that are bringing innovations to both the preclinical and clinical research arenas. In short, these are exciting times for biomedical research.

While past successes and our fervent hope for future ones surely help to explain the high status that the public confers on research, if our task here is to inquire about the overall trustworthiness of the system, then we also need to examine the biomedical research endeavor in aggregate and not just its star achievements. When we do, we quickly discover that there are many aggregate findings about research that place the foregoing inspiring features of biomedical research in a sobering context.

For example, it is estimated that as much as 85% of the resources consumed by both preclinical and clinical research are wasted. They are wasted not because the research fails to eventually produce new drugs, recalling our earlier admonition that would be an unreasonable expectation to place upon research. They are wasted instead because the research ecosystem itself is structurally deficient. Too often both preclinical and clinical studies are poorly designed, erroneously analyzed, inadequately and often misleadingly disseminated, and/or redundant (Chalmers and Glasziou, 2009; Paul Glasziou and Chalmers, 2016).

When we descend a bit from the 35,000 foot level and look specifically at aggregate findings about clinical trials for new drugs, we find that approximately 90% of the ones that enter Phase 1 trials fail to progress to regulatory approval,^2^ while about half of Phase II and III clinical trials fail because of lack of efficacy, and another quarter fail because of safety concerns (Harrison, 2016). Compounding matters further is the fact that the chances are high that much, and possibly even all, of the knowledge produced by the trials, and thus the benefit gained by conducting them, will not ever be known by others because, since the results very likely will never be published in full or possibly even in part, the potential knowledge lurking in the information produced by the studies simply will not be discoverable (Riveros et al., 2013; Goldacre et al., 2018). In other words, even though the research produces information that could contribute to the knowledge ecosystem, it gets withheld from it.

When we look to aggregate findings about the new drugs that do make it all the way through to approval, typically around 75% of them in any given year fail to perform any better than previously approved ones (Relman and Angell, 2002; New drugs and indications in 2011, 2012; PRESCRIRE AWARDS, 2019). Furthermore, for some of the new ones that do get approved, they get approved on the basis of being less effective than other drugs already proven to be effective (Powers and Fleming, 2013; Powers et al., 2018; Palmas, 2017). Many of these trials are known, bewilderingly, as non-inferiority trials even though they often seek the approval of therapeutically inferior drugs. Such studies seek to determine whether this inferiority is reasonably offset by ancillary benefits, such as more convenient dosing or different and potentially less burdensome side effects, compared to the other approved drugs that they are meant to replace for a patient’s clinical care. If they are to be ethical, though, the endpoints built into the studies meant to assess whether tolerating a “non-inferior” product is warranted must be justified. Evidence disturbingly shows that too often the comparative margins used to make the assessments are either unreported or poorly justified (Bai et al., 2021). The end result is that the sacrifices made by the volunteers who make the clinical trials possible, frequently, it should be noted, through woefully unethical informed consent processes (Doshi et al., 2017), can get wasted in terms of producing reliable information that can accurately inform future clinical treatment decisions (Powers and Fleming, 2013; Powers et al., 2018).

Aggregate findings further show that just because a new drug wins approval, there is no guarantee that it is truly either safe or effective for treating the populations of patients it is approved for. For example, between the years of 1965 and 2011, just under 50 analgesics were withdrawn post-licensure in multiple jurisdictions around the world due to adverse reactions (Onakpoya et al., 2017). Similarly, between the years of 1964 and 2009, 25 anti-obesity medications were withdrawn due to adverse reactions (Onakpoya et al., 2016). Countering these alarming numbers of withdrawals is the fact that, since the 1960s, the rate of withdrawal of approved drugs due to safety concerns has remained both low and steady, at around 2% (Rawson, 2016).

However, we ought not conclude from that consistently low rate that the remaining approved drugs not withdrawn from the market are therefore safe and effective. To understand why, we must factor in the reliability of post-marketing reporting of adverse events. Physicians are supposed to report adverse events to sponsors, who in the United States are supposed to report them to the FDA, which is supposed to compile and review the reports and, when warranted, withdraw approvals. Far too often this monitoring system is cumbersome, slow, and ineffective (Spencer et al., 2016). Its ineffectiveness means that information, if it were properly gathered and analyzed would lead to withdrawing a drug from the market, goes unreported and thus unconsidered instead, which means that patients continue to use many dangerous drugs and devices.

Other aggregate findings alert us to the presence of bad actors, principally clinical trial sponsors, who have been shown to both suppress safety and other data that, when not suppressed, could jeopardize a new drug’s chances of receiving regulatory approval. Those same sponsors then aggressively market the drugs despite the suppressed data they are in possession of. Probably the best poster children of such instances are the infamous cases of Vioxx used to treat arthritis and acute pain, Paxil used to treat depression and anxiety, and Zoloft also used to treat depression and anxiety, where data about fatal side effects of the drugs were suppressed (The Lancet, 2004; Lemmens, 2004).

While there is a temptation to believe that such instances of willful suppression of data and/or aggressive marketing of bad drugs are rare, that is a temptation that the facts belie. Far too often marketing considerations trump good science in industry funded research, so there is that to keep in mind (Fiacco et al., 2015).^3^ Also, we must factor in the lax approach that drug regulators, such as the FDA, take toward oversight of clinical trials and the bad actors that can sabotage them. For example, a recent analysis of FDA clinical trials oversight found that the agency rarely takes corrective action and, when it does, it often favors “voluntary actions” on the part of researchers or sponsors when it thinks corrective actions are needed. (Piller, C. (n. d.)).

Such worrisome corporate behavior and regulatory agency laxity toward it are particularly concerning when you consider that the public is already jaded toward pharmaceutical companies. The United States polling firm Gallup routinely polls public opinion about major industries and reports that “[t]he pharmaceutical industry is now the most poorly regarded industry in Americans’ eyes, ranking last on a list of 25 industries that Gallup tests annually.” (Inc G. Gallup.com, 2019) Such low trust no doubt reveals public unease with the monetization of research.

This unease is worth noting in regard to universities as well as drug companies, due in large part to passage of the Bayh-Dole Act. Enacted in 1980, it made it possible for universities to retain ownership of, and thus profit from, discoveries that resulted from federally funded research. Consequently, universities now support their research missions not just because they value discovery. They also routinely seek to monetize their research (Elizabeth Popp Berman, 2012). This means that the financial conflicts of interest that bedevils so much industry sponsored drug research can similarly taint university discovery missions as well (Yarborough, 2021b).

There is one final aggregate finding to note. It is the fact that every clinical trial, including all the worrisome ones alluded to above, share two things in common: they all receive IRB or similar approval and all of the countless people who volunteer to participate in them, and who thus make them possible to conduct, give their informed consent in order to be in them. Hence, IRBs perform poorly when it comes to weeding out uninformative clinical trials (Yarborough, 2020a) or assuring that informed consent processes convey all the information that is relevant to making informed decisions about trial participation (Yarborough, 2020b).

The problems discussed here are not highlighted to cast biomedical research in a bad light. Instead, they are highlighted to juxtapose the public trust that helps to fuel biomedical research with an objective picture of the research that trust makes possible. What emerges from that juxtaposition is a persistent theme throughout the essay, which is that the mechanisms found in Figure 2 that we currently rely upon to assure the trustworthiness of research are weak and ineffective far more often than people likely believe. This matters deeply if we care deeply, and almost all of us do, about biomedical research. How, then, might we better assure its trustworthiness?

## Where might we turn for guidance to move toward a more trustworthy system?

The prospects for transitioning to a biomedical research endeavor that will be more likely to repay the public’s trust in it with research that routinely deserves that trust will require multiple changes across an array of aspects that impact the culture and outcomes of research. Space does not permit anything other than a brief exploration of three research-impacting domains where a plethora of changes are needed: targeted reforms, better metrics, and proper diagnostics.

### Targeted reforms

If we want to increase trustworthiness, efforts are needed that combat the production and dissemination of undependable research results. Fortunately, there have been a host of reforms proposed for improving both the culture and work of biomedical research that, if enacted, could help to increase the dependability of published findings. The metaresearch (Ioannidis, 2018) community has been at the forefront of most of these efforts. Some representative publications from it bear particular mention. First is a series of articles that appeared a decade ago in the journal *Lancet* that discussed critical matters that could contribute to increasing value and reducing waste in research (Macleod et al., 2014), is the lead article in the series. The articles in the series reflect the broad scope of efforts required. Next is another publication notable for its comprehensive approach to the challenge of improving biomedical research, “A Manifesto for Reproducible Science.” (Munafò et al., 2017) These particular publications are highlighted not because they are definitive but because they capture the kinds of problems that need to be addressed, much of the impact of those problems, and specific reforms that can be adopted to combat them. The bulk of the reforms are intended to increase the use of appropriate study design and more robust reporting of research results so that the dependability and reproducibility of research will be enhanced. The challenge, as with all reform efforts, is how to achieve adoption of the recommended reforms, especially in the face of entrenched beliefs in the biomedical research community that diminishes receptivity to recommended reforms (Yarborough et al., 2019).

Also of note are a series of international conferences hosted by The World Conferences on Research Integrity Foundation (ID22, 2025). These conferences produce important guidance documents, nurture an international cohort of scholars of research integrity, which is vital to trustworthy research, and create venues for the exchange of ideas and dissemination of research results that relate to research integrity. Hence, the conferences are important resources that can help to overcome at least some of the inertia that hinders reform efforts that could help assure a trustworthy biomedical research endeavor.

### Better metrics

The biomedical research endeavor is largely shaped by, if not a slave to, key metrics. Probably the two most influential ones are drug and device marketing for companies and professional success for academic and other investigators. A necessary condition for most marketing is government licensure while professional success and the job protection it affords, at least for most academic researchers, turns largely on publication and funding. These prominent metrics reveal critical vulnerabilities to trustworthiness. What if licensure is only loosely, not rigorously, tied to actually safe and effective drugs and devices? What if publication and funding is not directly tied to the potential for producing and disseminating trustworthy research results? In both instances you get a biomedical research endeavor that is too prone to conducting and disseminating untrustworthy research.

This suggests that better metrics are needed to at least stand alongside, if not replace, the two aforementioned metrics. Multiple candidate metrics are implicit in the previously discussed transaction at the heart of the biomedical research endeavor. Let us look briefly at just one of them, which pertains to the academic institutions which house a large portion of biomedical researchers.

These institutions persistently tell the public how busy they are curing disease. But do they ever truly assess what progress they make toward that goal? If not, then they run the risk of saying one thing yet doing another, which is antithetical to efforts to deserve trust. So long as institutions only judge their faculties almost exclusively through the aforementioned metrics of publication and funding, they can never really know the extent to which they are moving toward their publicly stated goals because current faculty metrics are very poor secondary endpoints that too often have little if anything to do with the primary endpoint of curing disease^4^.

After all, one can have a long string of publications and funded research that has little if any impact in providing information relevant to better treatment or diagnostics. One should not infer from this that negative findings are not valuable because they often can be quite valuable. Rather, the larger point is that if we never bother to ask the question, have we as an institution moved the needle in any meaningful way in the past, say, five, ten, 15 years, toward the health outcomes we publicly tout, the answer could well be no. And chances are high that no one would take substantive note of lack of progress and thus be open to the need to look for correctives to the unpromising trajectory.

Something is needed to disrupt this unsettling picture, which supports this current recommendation to implement institutional metrics tied directly to the primary endpoints touted in institutional rhetoric about their research missions and their importance. If institutions would start to meaningfully hold themselves accountable to these lofty goals they tout to the public, this would do much, that currently is not being done, to improve the trustworthiness of research.^5^

### Proper diagnostics

The implementation of reforms and adoption of critical metrics are necessary if we want to better assure trustworthiness, but they are not sufficient. We also need a diagnostic tool or tools to help track their impact.

**FIGURE 4:**
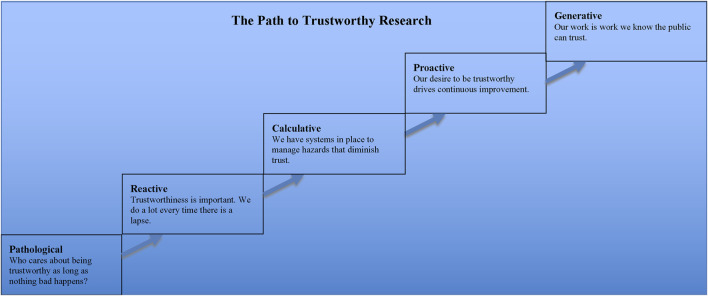
Legend The manner in which scientific research is conducted confers varying degrees of trustworthiness upon it. The farther along the path to trustworthiness a research group or institution is, the more practices there are that have been introduced for the specific purpose of reducing the number of lapses that diminish trust in research.

An article by Professor Patrick Hudson of Leiden University that contained a helpful graphic for describing the evolution of safety cultures in industrial settings provides insights about such tools (Hudson, 2003). Previously, colleagues and I adapted his graphic to the biomedical research community (Yarborough et al., 2009), and I further adapted it in a subsequent publication (Yarborough, 2014b). That latest version of the graphic is reproduced here as Figure 4. It illustrates how one can track progress, in both individual and institutional and even multi-institutional research settings, towards the production and dissemination of trustworthy research.

Looking at the graphic helps us to appreciate the shortsightedness of taking a reactive as opposed to proactive stance to the public’s trust in research. Although, as my colleagues and I pointed out in our article, it would be unfair to say that the research community is stuck at the pathological or reactive levels, it would be equally wrong to claim that it is anywhere near the desired proactive stage, let alone the generative one. Thus, there is much work that still needs to be done if the research community wants to reach the point wherein members of that community can be confident that “our work is work we know the public can trust.”

It is important to note one further item about that graphic, however, before moving on. The point is that generating interest in a path to trustworthiness and the steps along it will face a strong headwind. Many people both within and without the research community may very well believe that biomedical research is already at the generative stage because that is where all science, by definition, should be. I say that because science is science in large part because of how it is thought by most people to operate.

Recalling comments above about the characteristic of dependability, as an organized activity aimed at making discoveries about the natural world, science is presumed to exhibit methodological features meant to produce dependable findings. In other words, the activity itself should assure us about its quality and value because science, by its very nature, produces sound work. Hence, the very invocation of science blunts curiosity about its quality and value and thus obviates the need for further concern about where along the path to trustworthy research the research community, in whole or part, may be because the path does not apply to science.

The thrust of this essay is meant to show how erroneous it is to think that biomedical research, just because it is science, need not worry about the path and how far away it is from its generative stage. All the more reason, then, that we need to be vigilant about implicit assumptions we may be tempted to make about any built-in protections at work in science that insulates the research community from the need to make important and difficult transformations if it wants to routinely warrant the public’s trust.

## Conclusion

The foregoing has attempted to provide both insights into and some of the context for substantively considering whether or not Paul Gelsinger’s determination that the biomedical research system is not trustworthy is correct. To say it is incorrect would mean that readers or their loved ones could trust that a gene therapy or other clinical trial they may be asked to volunteer for is based on a body of dependable prior evidence showing that what is being investigated in the trial is actually worth investigating, the methods and protocol it employs are capable of producing dependable information, and that the trial will be conducted in a manner that treats volunteers with the full measure of respect that is their due.

No doubt, any clinical trial will be preceded by peer-reviewed publications, will credibly pursue information meant to pertain to improved diagnostics or therapies, and be IRB approved. Why, though, are they able to trust that enough of those publications have reported dependable results, that dependable information is capable of being produced by the trial, that the information is sought in pursuit of better, rather than just new, diagnostics or therapeutics, or that an IRB review is capable of ferreting out unethical clinical trials? I think the foregoing discussion has drawn on sufficient evidence showing that many, dare I say most, reasonable people would answer that question in the negative.

Bolstering this negative response are recent remarks by Stephen Rosenfeld, former chair of the US Secretary’s Advisory Committee on Human Research Protections (SACHARP). He is likely in as good a position as anyone to answer these closing questions. He chaired SACHARP from December 2016 to January 2021 and recently stated, more than 20 year’s after Mr. Gelsinger’s son’s death, that “we owe the public an accounting” for matters such as published studies that must be retracted and asking people to volunteer for uninformative clinical trials (JD Supra). I think his remarks should give pause to anyone who wants to claim the system is now trustworthy.

## Data Availability

The original contributions presented in the study are included in the article/supplementary material, further inquiries can be directed to the corresponding author.

## Footnotes

2 That a low percentage of therapeutics investigated in early phase trials eventually make it to the drug approval stage is certainly to be expected. However, studies suggest that we should rightly question that the rate should be so high. Early phase studies of serious neurological disorders are particularly noteworthy in this regard. For example, a study published in 2013 suggested that only 8 out of 160 early phase human trials were likely scientifically and ethically warranted, given quality concerns pertaining to vast numbers of the preclinical studies used to justify the human trials (Tsilidis et al., 2013). Similarly, another study published in 2020 raised the alarming prospect the majority of clinical trials for stroke were based on preclinical studies that reported what were most likely to be false positive findings (Schmidt‐Pogoda et al., 2020).

3 For a thoughtful exploration of the extent to which marketing shapes the clinical research of pharmaceutical companies I refer readers to the work of Sergio Sismondo, in particular his book about the topic (Sismondo, 2018).

4 It is worth noting the recent dramatic cuts to federal funding of biomedical research in the United States due to the actions of the presidency of Donald Trump. Whether the funding cuts will be temporary or long-lasting remains to be seen. However, it is likely the effect of the reductions will be significant. Research is already being halted and careers of current and future researchers are being either ended or significantly curtailed. The reasons for the dramatic funding cuts appear to be driven largely for political considerations with little to no connection with the quality considerations that this essay focuses on. I say this because the executive decisions were hastily made, apparently to punish universities and academic medical centers since the cuts were not made after any careful, principled, and impartial assessment of the overall state of research at those institutions to produce findings that could have sought to identify deficiencies in need of redress that funding cuts could plausibly address. Thus, one is left to speculate whether the executive actions will do anything at all to improve the trustworthiness of biomedical research. Furthermore, by politicizing federal support for research it is possible that the cuts will have the opposite effect. Biomedical research going forward may be perceived to lack the independence of thought that is needed if institutions and those who conduct research at them are to be able to pursue and conduct research capable of consistently carrying out socially valuable, dependable and ethical investigations.

5 Adoption of new metrics along the lines of the one proposed here obviously will face multiple fierce headwinds. Two deserve special mention. First of all, institutions like large universities rarely voluntarily take on extensive reform efforts that metrics like the one proposed here would require. Second, there is much about institutional culture, in particular prominent beliefs of both faculty members and administrators that inhabit them that disinclines entertainment of the need for the sort of proposed metric in the first place (Hossenfelder, 2017). First of all, many, probably most, academics chose their profession in large part because they want to make the world a better place. Second, there is a widespread, embedded confidence in the inevitable ameliorative effect, if not eventual triumph, of biomedical research over human disease. Both can serve as blinders that inhibit inquisitiveness about the need for having appropriate institutional accountability in place.
